# *Eucommia ulmoides* Oliv. (Du-Zhong) Lignans Inhibit Angiotensin II-Stimulated Proliferation by Affecting P21, P27, and Bax Expression in Rat Mesangial Cells

**DOI:** 10.1155/2015/987973

**Published:** 2015-06-10

**Authors:** Xian Jing, Wei-Hua Huang, Yong-Jun Tang, Ya-Qin Wang, Hui Li, Ying-Ying Tian, Yao Chen, Hong-Hao Zhou, Dong-Sheng Ouyang

**Affiliations:** ^1^Department of Clinical Pharmacology, Xiangya Hospital, Central South University, Changsha 410008, China; ^2^Institute of Clinical Pharmacology, Hunan Key Laboratory of Pharmacogenetics, Central South University, Changsha 410078, China; ^3^Yihe Hospital of Zhengzhou, Zhengzhou 450047, China

## Abstract

Cortex Eucommiae (*Du-zhong*) is the dried bark of the* Eucommia ulmoides *Oliv. The natural products identified from* Du-zhong* include lignans, iridoids, flavonoids, polysaccharides, terpenes, and proteins, Liu et al. (2012). Lignans, the main bioactive components, were protective against hypertensive renal injury in spontaneous hypertensive rats in our previous study, Li et al. (2012). Moreover,* Eucommia* lignans also diminished aldose reductase (AR) overexpression in the kidney, Li et al. (2012). However, the pathological mechanism underlying the protective effects of* Eucommia* lignans remains unknown. Cellular proliferation was reported to contribute to important pathological changes in hypertensive renal injuries, and increased angiotensin II (Ang II) expression was reported to be essential for target-organ damage during hypertension. Ang II is the main effective peptide in the renin-angiotensin system and is considered to be a key mediator in the development of hypertensive nephropathy, Rüster and Wolf (2011). Our preliminary results showed that* Eucommia* lignans had inhibitory effects on Ang II-induced proliferation of rat mesangial cells. In the present study, we investigated the effects of* Eucommia ulmoides *on Ang II-induced proliferation and apoptosis of rat mesangial cells. Cell cycle-related genes P21 and P27, and cell apoptosis-related genes Bax and Bcl-2, were determined.

## 1. Introduction

Cortex Eucommiae (*Du-zhong*) is the dried bark of the* Eucommia ulmoides* Oliv. (family: Eucommiaceae) and is one of the most popular tonic herbs in Asia. The natural products identified from* Du-zhong* include lignans, iridoids, flavonoids, polysaccharides, terpenes, and proteins [1]. Lignans, the main bioactive components, were protective against hypertensive renal injury in spontaneous hypertensive rats in our previous study [2]. Further study showed that* Eucommia* lignans decreased both N-acetyl-*β*-D-glucosaminidase enzyme activity and the ratio of albumin to urinary creatinine in spontaneous hypertensive rats. Moreover,* Eucommia* lignans also diminished aldose reductase (AR) overexpression in the kidney [2]. However, the pathological mechanism underlying the protective effects of* Eucommia* lignans remains unknown. Cellular proliferation was reported to contribute to important pathological changes in hypertensive renal injuries, and increased angiotensin II (Ang II) expression was reported to be essential for target-organ damage during hypertension. Ang II is the main effective peptide in the renin-angiotensin system and is considered to be a key mediator in the development of hypertensive nephropathy [3]. Ang II regulates the proliferation of RMCs and increases the formation of the extracellular matrix primarily through the induction of glomerular hypertension and nonhemodynamic effects, including the production of reactive oxygen species and the upregulation of fibrotic growth factors. Our preliminary results showed that* Eucommia* lignans had inhibitory effects on Ang II-induced proliferation of rat mesangial cells. In the present study, we investigated the effects of* Eucommia ulmoides* on Ang II-induced proliferation and apoptosis of rat mesangial cells.

## 2. Materials and Methods

### 2.1. Materials and Reagents

Human angiotensin II was purchased from Sigma-Aldrich (St. Louis, MO, USA). The lignan content in* Eucommia* lignans was determined to be 71% by spectrophotometry on a Beckman Coulter DU 640 spectrophotometer (Beckman Coulter, Inc., USA) at 277 nm; the lignans were extracted from* Eucommia ulmoides* Oliv. bark in our own laboratory using a previously described method [2].

The primers for P21, P27, Bax, Bcl-2, and AR were purchased from Invitrogen (Carlsbad, CA, USA). Antibodies against P21, P27, Bax, Bcl-2, and AR for western blotting were supplied by Abcam (Cambridge, England) and Santa Cruz (CA, USA). The Cell Titer 96 Aqueous One Solution Proliferation Assay for the 3-(4,5-dimethylthiazol-2-yl)-2, 5-diphenyl tetrazolium bromide (MTT) method was purchased from Promega (Madison, WI, USA). The RT-qPCR kits with Platinum SYBR Green qPCR Super Mix-UDG and flow cytometry were purchased from Invitrogen (Carlsbad, CA, USA). Epalrestat was obtained commercially from Yuancheng Pharmaceutical Co., Ltd. (Hubei, China). The MTT One-Step Cell Proliferation Test Kit and Annexin V-FITC Apoptosis Assay Kit were purchased from the Promega Corporation and Bibo Biological Technology Co., Ltd. (Nanjing, China), respectively.

### 2.2. Cell Culture

Rat mesangial cells (RMCs, China Type Culture Collection, Wuhan, China) were maintained in a humidified incubator with 5% CO_2_/95% air at 37°C and propagated in RPMI-1640 medium supplemented with 10% newborn calf serum (NCS). Cell lines were passaged every three days after treatment with trypsin-ethylenediaminetetraacetic acid. Cells were used for experiments from passages 5–15.

### 2.3. MTT Assay

RMCs viability was assessed by 3-(4,5-dimethylthiazol-2-yl)-2, 5-diphenyl tetrazolium bromide. Briefly, cells were plated in 96-well tissue culture plates at a density of 1 × 10^5^ cells/mL. The cells were washed twice with phosphate-buffered saline when they reached 60% confluence and incubated for 24, 48, or 72 h in serum-deprived medium. During this final incubation, RMCs were treated with one of the following regimens for 24, 36, or 48 hours: control group (cultured RMCs coincubated with 1% NCS medium), Ang II group (10^−8^ mol/L Ang II), epalrestat group (10^−8^ mol/L Ang II + 20 *μ*mol/L of the AR inhibitor epalrestat; positive control group), low concentration lignan group (10^−8 ^mol/L Ang II + 20 mg/L lignans), middle concentration lignan group (10^−8^ mol/L Ang II + 40 mg/L lignans), and high concentration lignan group (10^−8^ mol/L Ang II + 80 mg/L lignans). Each concentration was tested in at least six replicates. Subsequently, 20 *μ*L of the Cell Titer 96 Aqueous One Solution Reagent was added to each well, and the plates were incubated at 37°C for an additional 2 h. The absorbance of the solubilized blue formazan at 490 nm was recorded using a microplate reader (Beckman Coulter, Inc., USA).

### 2.4. Reverse Transcription Real-Time Quantitative PCR (RT-qPCR) Assay

RMCs were seeded into six-well plates at a density of 1 × 10^5^ cells per well. The 6 groups [control group (cultured RMCs coincubated with 1% NCS medium), Ang II group (10^−8^ mol/L Ang II), epalrestat group (10^−8^ mol/L Ang II + 20 *μ*mol/L epalrestat), and the lignan groups (10^−8^ mol/L Ang II + 20, 40, or 80 mg/L lignans)] were cultured in RPMI-1640 medium containing 1% NCS for 48 h. The total RNA from the RMCs was extracted using the TRIzol reagent (Invitrogen, CA, USA), and the RNA concentration was determined by spectrophotometry at 260 and 280 nm. The cDNA was reverse-transcribed from the total RNA and treated with the real-time RT-PCR kits. The gene-specific primers are listed in Table 1. The data were quantitatively analyzed with the Stratagene Mx3000p Real-Time PCR machine (Santa Clara, CA, USA). The beta-actin (*β*-actin) and glyceraldehyde phosphate dehydrogenase (GAPDH) genes were used as internal controls.

### 2.5. Western Blot Assay

Total protein was extracted from RMCs with radioimmunoprecipitation assay lysis buffer after 48 h of culture under the conditions described above; the protein concentration was determined with a Bicinchoninic Acid Assay Kit (Sigma-Aldrich, USA). Samples containing 40 *μ*g of total protein were separated on a 10% sodium dodecyl sulfate polyacrylamide gel and transferred onto a polyvinylidene fluoride membrane. The membrane was blocked with 5% skim milk solution in Tris-buffered saline supplemented with 0.1% Tween-20 over night. Subsequently, one of the primary antibodies (rabbit polyclonal antibody to P21, goat polyclonal antibody to P27, rabbit polyclonal antibody to Bax, mouse monoclonal [IST-9] antibody to Bcl-2, goat polyclonal antibody to AR, or rabbit polyclonal antibody to GAPDH) was added for hybridization. Then, the blots were incubated with the specific secondary antibody after the membranes were washed with TBST three times. Finally, the protein bands were visualized using the Enhanced Chemiluminescence Western Blotting Detection System (Millipore, USA), and *β*-actin and GAPDH were selected as the internal standards for data normalization.

### 2.6. Cell Cycle and Apoptosis Analysis

Rat mesangial cells were treated with one of the following regimens for 24, 36, or 48 h: (1) control; (2) Ang II group (10^−8^ mol/L Ang II); (3) epalrestat group (10^−8^ mol/L Ang II + 20 *μ*mol/L epalrestat); and (4) Ang II + different concentrations of lignans (20, 40, or 80 mg/L). Each concentration was tested with at least three replicates. Briefly, 4 × 10^4^ cells were harvested by centrifugation, washed with phosphate-buffered saline, fixed with 70% (v/v) cold aqueous ethanol, and stored overnight at −20°C. The fixed cells were washed with phosphate-buffered saline and incubated with propidium iodide containing 0.05% RNase. The samples were incubated at 4°C in the dark and analyzed by flow cytometry. The percentage of cells in the S, G_0_, G_1_, G_2_, and M phases was analyzed using Diva software (Data-Interpolating Variational Analysis). Also apoptosis rate (%) = number of apoptotic cells/total cells × 100%.

### 2.7. Enzyme Activity Assays

After the cells were cultured in 6-well plates for 48 h, nonadherent cells were extracted into a 1.5 mL EP tube. To recover the adherent cells, 500 *μ*L of trypsin (0.25%) was added to the 6-well plates and incubated for approximately 30 s; then, the nonadherent cells and adherent cells were mixed and centrifuged at 1000 r/min for 10 min and washed twice with PBS. A total of 200 *μ*L of potassium phosphate buffer (pH 6.0) was added to the 1.5 mL EP tubes to resuspend the precipitates, and then the precipitates were shaken in a humidified incubator at 4°C for 10 min. The tube contents were mixed by flicking with a finger to ensure perfect cell lysis. No obvious cell precipitation was observed after full lysis. Then, the cells were centrifuged at 12,000 r/min for 40 min, and the supernatant was used to assess the enzyme activity.

Each sample was incubated in triplicate on ice. The reaction system (200 *μ*L) consisted of 5 *μ*L of supernatant, 135 mM PBS, 100 mM ammonium sulfate, 0.04 mM DL-glyceraldehyde, and 150 *μ*M NADPH. The reaction was kept in a humidified incubator at 37°C for 4 min after the addition of DL-glyceraldehyde. The absorbency was recorded using an ultraviolet spectrophotometer (Xianke Instrument Co., Ltd., Shanghai, China).

### 2.8. Statistical Analysis

Data were expressed as the mean ± SD. The differences between groups were analyzed by one-way analysis of variance and the Student-Newman-Keuls *t*-test with the SPSS 17.0 software. Statistical significance was defined as *P* < 0.05.

## 3. Results

### 3.1. Effects of* Eucommia* Lignans on Ang II-Induced Changes in the RMCs Cell Cycle

The cell cycle distribution of RMCs treated with Ang II showed a significant reduction in the ratio of cells in G_1_ phase (*P* < 0.05) and an elevation in the ratio of cells in S phase. These effects were alleviated by* Eucommia* lignans (*P* < 0.01) (Table 2) in a concentration-dependent manner.

### 3.2. Effects of* Eucommia* Lignans on Ang II-Induced P21 and P27 Expression in Rat Mesangial Cells

**Figure 1:**
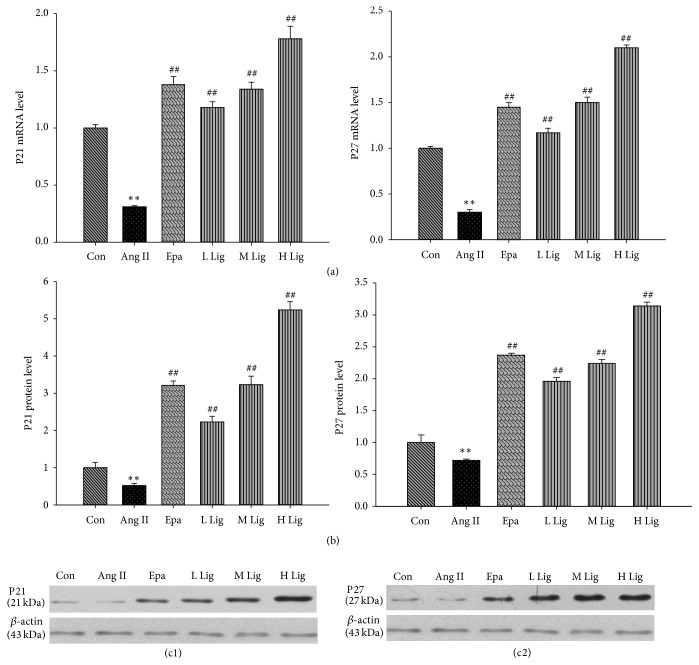
Effect of lignans on expression of P21 and P27 in RMCs induced by Ang II. *β*-actin and GAPDH were used as an internal loading control gene. Both mRNA and protein relative expression levels were expressed as folds of control. All values were expressed as mean ± SD (*n* = 3). (a) Effect of lignans on mRNA expression of P21 and P27 by RT-qPCR. (b, c1~c2) Effect of lignans on protein expression of P21 and P27 by western blot; Con: control; Ang II: 10^−8^ mol/L Ang II group; Epa: 10^−8^ mol/L Ang II + 20 *μ*mol/L epalrestat group; L Lig: 10^−8^ mol/L Ang II + 20 mg/L lignan group; M Lig: 10^−8^ mol/L Ang II + 40 mg/L lignan group; H Lig: 10^−8^ mol/L Ang II + 80 mg/L lignan group; ^*∗*^ *P* < 0.05, ^*∗∗*^ *P* < 0.01 compared with the control group; ^#^ *P* < 0.05, ^##^ *P* < 0.01, compared with the 10^−8^ mol/L Ang II group.

The RT-qPCR assay showed that the P21 and P27 mRNA expression levels in the Ang II group were weaker than the control (*P* < 0.01) (Figure 1). Western blot analyses demonstrated that P21 and P27 protein expression levels in the Ang II group were weaker than the control (*P* < 0.01). However, the mRNA and protein expression of P21 and P27 were significantly increased by the* Eucommia* lignans (20, 40, and 80 mg/L) (Figure 1).

### 3.3. Effects of* Eucommia* Lignans on Ang II-Induced Cellular Apoptosis in RMCs

**Figure 2:**
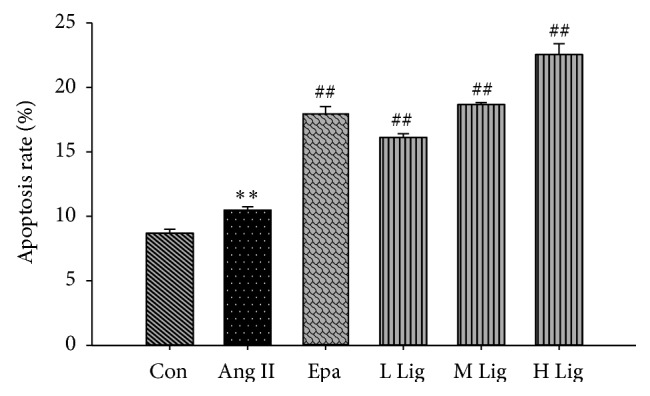
Effect of lignans on cell apoptosis rate in RMCs induced by Ang II. All values represented mean ± SD (*n* = 3). Con: control; Ang II: 10^−8^ mol/L Ang II group; Epa: 10^−8^ mol/L Ang II + 20 *μ*mol/L epalrestat group; L Lig: 10^−8^ mol/L Ang II + 20 mg/L lignan group; M Lig: 10^−8^ mol/L Ang II + 40 mg/L lignan group; H Lig: 10^−8^ mol/L Ang II + 80 mg/L lignan group. ^*∗*^ *P* < 0.05, ^*∗∗*^ *P* < 0.01 compared with the control group; ^#^ *P* < 0.05, ^##^ *P* < 0.01, compared with the 10^−8^ mol/L Ang II group.

The apoptosis rates in the Ang II group showed a slight increase compared with the control group (Figure 2). Furthermore, the apoptotic rates in the* Eucommia* lignans groups (20, 40, and 80 mg/L) were further increased compared with the Ang II group.

### 3.4. Effects of* Eucommia* Lignans on Ang II-Induced Bax and Bcl-2 Expression in Rat Mesangial Cells

**Figure 3:**
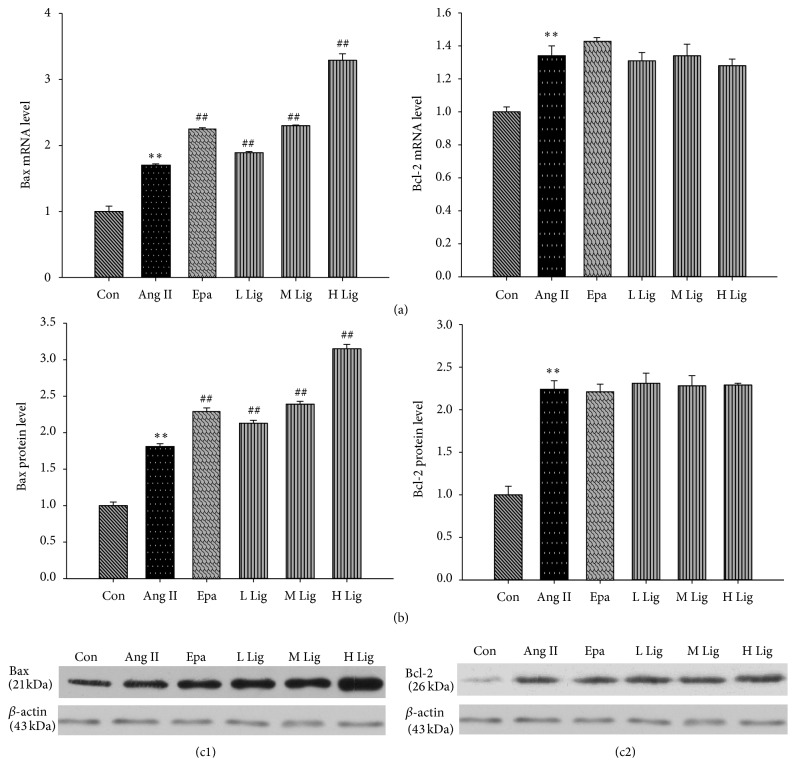
Effect of lignans on expression of Bax and Bcl-2 in RMCs induced by Ang II. *β*-actin and GAPDH were used as an internal loading control gene. Both mRNA and protein relative expression levels were expressed as folds of control. All values were expressed as mean ± SD (*n* = 3). (a) Effect of lignans on mRNA expression of Bax and Bcl-2 by RT-qPCR. (b, c1~c2) Effect of lignans on protein expression of Bax and Bcl-2 by western blot. Con: control; Ang II: 10^−8^ mol/L Ang II group; Epa: 10^−8^ mol/L Ang II + 20 *μ*mol/L epalrestat group; L Lig: 10^−8^ mol/L Ang II + 20 mg/L lignan group; M Lig: 10^−8^ mol/L Ang II + 40 mg/L lignan group; H Lig: 10^−8^ mol/L Ang II + 80 mg/L lignan group; ^*∗*^ *P* < 0.05, ^*∗∗*^ *P* < 0.01 compared with the control group; ^#^ *P* < 0.05, ^##^ *P* < 0.01, compared with the 10^−8^ mol/L Ang II group.

Bax mRNA and protein expression levels were stronger in the Ang II group (Figure 3). Moreover, Bax expression was increased in the* Eucommia* lignans groups compared with the Ang II group. In contrast, there was no significant difference in Bcl-2 mRNA and protein expression between the* Eucommia* lignans groups and the Ang II group (*P* > 0.5).

### 3.5. Effects of* Eucommia* Lignans on Ang II-Induced AR in RMCs

**Figure 4:**
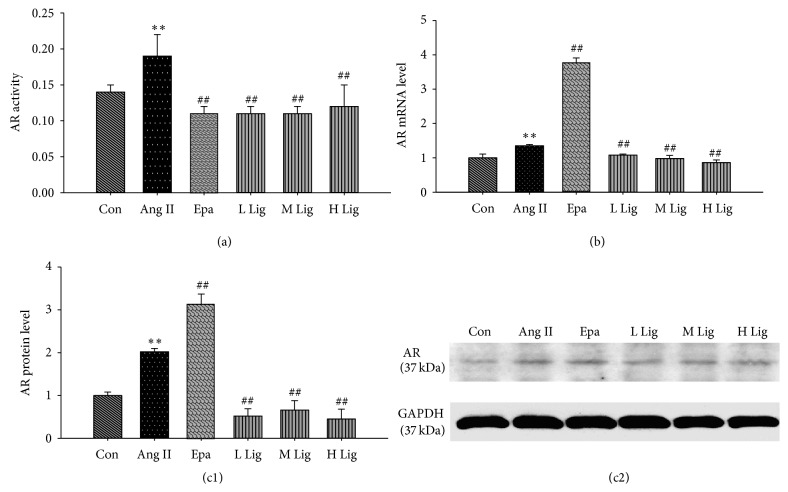
Effect of lignans on AR in RMCs induced by Ang II. GAPDH was used as an internal loading control gene. All values were expressed as mean ± SD (*n* = 3). (a) Effect of lignans on activity of AR; (b) effect of lignans on mRNA expression of AR by RT-qPCR; (c1, c2) effect of lignans on protein expression of AR by western blot. Con: control; Ang II: 10^−8^ mol/L Ang II group; Epa: 10^−8^ mol/L Ang II + 20 *μ*mol/L epalrestat group; L Lig: 10^−8^ mol/L Ang II+ 20 mg/L lignan group; M Lig: 10^−8^ mol/L Ang II + 40 mg/L lignan group; H Lig: 10^−8^ mol/L Ang II + 80 mg/L lignan group; ^*∗*^ *P* < 0.05, ^*∗∗*^ *P* < 0.01 compared with the control group; ^#^ *P* < 0.05, ^##^ *P* < 0.01, compared with the 10^−8^ mol/L Ang II group.

The mRNA, protein expression, and activity of AR were effectively enhanced by Ang II (Figure 4).* Eucommia* lignans suppressed Ang II-induced AR expression in RMCs.

## 4. Discussions

Mesangial cells are the targets of bioactive factors (i.e., a variety of immune complexes, cytokines, thrombin, and integrins). These factors have a variety of physiological functions, such as the secretion of extracellular matrix, devouring, shrinkage, and maintaining normal substrate metabolism [4]. However, RMCs show significant proliferation, which is the core pathological aspect of a variety of kidney diseases. In our previous study, rat mesangial cells treated with Ang II showed significant cell proliferation compared with the control group (*P* < 0.01) [2]; the* Eucommia* lignans effectively inhibited the proliferation of mesangial cells induced by Ang II. The* Eucommia* lignans were able to postpone glomerular sclerosis and prevent the deterioration of renal function [5]. The process of cell proliferation is an evolutionary process controlled by the cell cycle. A significant reduction in the ratio of cells in G_1_ phase and significant elevation in the ratio of cells in S phase indicate cells in a state of proliferation.* Eucommia* lignans significantly increased the ratio of cells in G_1_ phase and decreased the ratio of cells in S phase during the inhibition of proliferation. Thus, we concluded that the* Eucommia* lignans inhibited cell proliferation by acting on the cell cycle.

Consequently, we studied the cell cycle by analyzing the expression levels of the P21 and P27 genes. P21 is a negative regulator of the cell cycle, and elevated P21 expression prevents cells from transitioning from G_1_ to S phase [6, 7]. P27 is a pivotal negative regulatory protein in G_1_, and its expression level critically influences renal cell proliferation [8, 9]. Therefore, we measured the P21 and P27 mRNA and protein levels and found that P21 and P27 mRNA and protein expression in RMCs treated with Ang II was weaker, resulting in abnormal cell proliferation. The* Eucommia* lignans significantly increased the P21 and P27 mRNA and protein expression inhibited by Ang II. Thus,* Eucommia* lignans inhibited cell proliferation by acting on the cell cycle-related genes P21 and P27.

Maintaining the balance between cell proliferation and apoptosis is important for normal development and tissue homeostasis [10]. We assessed the apoptosis rates because apoptosis plays a critical role in the development of kidney disease [11]. The apoptosis rates in cells incubated with different concentrations of* Eucommia* lignans were higher compared to cells in the Ang II group. Based on these data,* Eucommia* lignans played an important role in promoting apoptosis.

Apoptosis is a complex process involving more than one gene [12]. Cell apoptosis plays a key role in renal disease outcome. Activated and proliferating RMCs can be eliminated through apoptosis, allowing the return of the normal structure and function of the glomerular [13]. Bcl-2 and Bax are two important genes involved in the apoptosis process. Bcl-2 is an antiapoptotic gene that belongs to a class of genes that inhibits apoptosis and can prevent the induction of apoptosis by a variety of factors. Indeed, excessive Bcl-2 expression can block the occurrence of apoptosis [14, 15]. Bax is a proapoptotic gene. Yamagishi et al. reported that apoptosis in diabetic nephropathy was related to Bax [16]. Our study showed that the* Eucommia* lignans significantly increased Bax expression and reduced the Bcl-2/Bax ratio. However, no obvious changes in Bcl-2 expression were observed in the present experiments. Based on these results, we speculated that the Ang II receptor type 2 activated phosphatase mitogen-activated protein kinase-1, resulting in the dephosphorylation of the Bcl-2 molecule and the loss of its antiapoptotic function [17]. Therefore, the change in Bax/Bcl-2 eventually resulted in apoptosis, and the* Eucommia* lignans promoted apoptosis by increasing Bax expression.

We also investigated apoptosis related to caspase-3 gene expression [18–20]; however, the results were not statistically significant.

AR, an important member of the aldehyde ketone reductase superfamily, is a rate-limiting enzyme in the polyol pathway involved in glucose metabolism [21]. AR is involved in the proliferation of mesangial cells, inflammation, apoptosis, tubular epithelial cell transition, and extracellular matrix deposition, which can induce hypertensive renal injury [22, 23]. Our previous study demonstrated that AR played an important role in the pathological processes of hypertension and hypertensive organ injury [2, 21]. Therefore, we concluded that high expression of AR may have a causal relationship with renal injury. As shown in this study, AR expression was much higher in the Ang II group compared to the control group and was highest in the epalrestat group; in contrast, AR expression was significantly reduced in the groups treated with different concentrations of lignans compared to the Ang II group. One possible reason may be that AR was inhibited by epalrestat; when the level of activated AR was reduced sufficiently to impair the maintenance of the normal physiological functions of RMCs, the cells began to increase AR expression. However, the specific mechanism requires further research.

In conclusion, the results of this study indicated that Ang II induced the proliferation and apoptosis of RMCs by decreasing the expression of P21, P27, and Bax.* Eucommia* lignans may play a protective role in the kidneys by inhibiting proliferation and promoting apoptosis through the upregulation of P21, P27, and Bax expression. The apoptosis related gene Bcl-2 showed no obvious effect in our study. This study provides convincing evidence for the use of* Eucommia* lignans in the therapy of kidney diseases.

## 5. Conclusions

*Eucommia* lignans are able to suppress the proliferation and promote the apoptosis of rat glomerular mesangial cells induced by Ang II by upregulating P21, P27, and Bax (but not Bcl-2) expression; AR may play a key role in this process.

## Figures and Tables

**Table 1:** Information on the primers used for real-time PCR.

Genes	Primers sequence
P21	F: 5′-TCATGGCGAGCTGTCTCCAG-3′
	R: 5′-CCCAGACGTAGTTGCCCTCC-3′

P27	F: 5′-GAGCTTGGATGTCAGCGGGA-3′
	R: 5′-CCGGTCCTCAGAGTTTGCCT-3′

Bax	F: 5′-GAAGATGGGCTGAGGCTTCCT-3′
	R: 5′-TTCCCCGTTCCCCATTCATCC-3′

Bcl-2	F: 5′-ATGTGTGTGGGGAGCGTCAA-3′
	R: 5′-GTGTGCAGATGCCGGTTCAG-3′

AR	F: 5′-TCCCAGGATCAAGGAAATTG-3′
	R: 5′-ACAACAGGAACTGGAGGGTG-3′

*β*-actin	F: 5′-CATTGTCACCAACTGGGACGATA-3′
	R: 5′-GGATGGCTACGTACATGGCTG-3′

**Table 2:** Effect of lignans on the cell cycle in RMCs induced by Ang II.

Groups	G1 period (%)	G2 period (%)	S period (%)
Con	73.9 ± 0.6	13.5 ± 0.7	12.6 ± 0.2
Ang II	69.2 ± 0.3^*∗*^	8.4 ± 0.8^*∗∗*^	22.4 ± 1.1^*∗∗*^
Epa	76.4 ± 1.4	12.5 ± 1.1^#^	11.1 ± 0.4^##^
L Lig	73.5 ± 2.5	12.5 ± 2.2^#^	14.0 ± 0.6^##^
M Lig	76.9 ± 4.1	13.0 ± 4.0^#^	10.1 ± 0.2^##^
H Lig	77.6 ± 0.7^##^	15.3 ± 1.3^##^	7.1 ± 0.7^##^

Note: RMCs were treated with Ang II (10^−8^ mol/L), epalrestat (20 *μ*mol/L), and various concentrations of *Eucommia* lignans (20, 40, and 80 mg/L) for 48 h, and cell cycle was assessed by flow cytometry. Results were given in mean ± SD (*n* = 3). ^*∗*^ *P* < 0.05, ^*∗∗*^ *P* < 0.01 compared with the control group; ^#^ *P* < 0.05, ^##^ *P* < 0.01, compared with the 10^−8^ mol/L Ang II group.

## Abbreviations

Ang II: Angiotensin II
RMCs: Rat mesangial cells
AR: Aldose reductase
NCS: Newborn calf serum
MTT: 3-(4,5-Dimethylthiazol-2-yl)-2, 5-diphenyl tetrazolium bromide.
